# Dengue’s Clinical Course in One of Colombia’s Most Endemic Areas: Impact of Endemic and Epidemic Periods

**DOI:** 10.4269/ajtmh.25-0158

**Published:** 2026-02-26

**Authors:** Gisel Viviana Osorio-Cuéllar, Juliana Quintero, Alvaro Andres Gaitan, Carlos Torres-Martínez, Marcela Daza, Gustavo Clemen, Luz Elena Muñoz Garzón, José Oñate, Mauricio Bernal-Sanchez, Martín Cañón Muñoz

**Affiliations:** ^1^ Research Center, Clínica Imbanaco, Cali, Colombia;; ^2^ Population Health, Fundación Santa Fe de Bogotá, Bogotá, Colombia;; ^3^ Takeda Colombia, Bogotá, Colombia;; ^4^ Universidad del Bosque, Bogotá, Colombia;; ^5^ Cafettor Médica S.A.S, Bogotá, Colombia;; ^6^ Hospital Universitario Evaristo García E.S.E, Cali, Colombia;; ^7^ Red de Salud Ladera E.S.E, Cali, Colombia;; ^8^ Clínica Imbanaco, Cali, Colombia;; ^9^ Clínica de Occidente, Cali, Colombia;; ^10^ Clínica Sebastián de Belalcázar, Cali, Colombia

## Abstract

Dengue is a viral disease with a broad spectrum of clinical manifestations. Its severity is associated with risk factors such as periods of endemicity, although the extent of this contribution is unclear. In the present study, the characteristics of the clinical course and differences in the occurrence of severe and lethal outcomes were assessed by the degree of endemicity in patients with dengue treated in health institutions in Cali, Colombia, between 2019 and 2022. A longitudinal, observational-historical, single-cohort study was conducted. Analysis involved evaluating clinical complications during epidemic and endemic periods. Mortality, cumulative incidence of hospital or intensive care unit (ICU) admission, and survival time were estimated using the competing risks method. A total of 1,038 dengue cases were included. Patients with severe dengue and those with warning signs (WS) were consulted up to 6 or 5 days after symptom onset, respectively. A hospital stay of 2.0 days (Quartile [Q]1–Q3: 2–4 days) and an ICU stay of 4.5 days (SD ± 3.9 days) were estimated. A mortality rate of 5.5% was obtained for severe dengue, and a mortality rate of 0.4% was identified for patients with WS. In 2020 and 2021, the probability of hospitalization was 20% from initial contact. The likelihood of dying within 10 days after initial contact was 0% in 2019 and 11.2% (95% CI: 4.0–22.5%) in 2020. The incidence of hospitalization, number of hospital days, and ICU stays due to dengue varied by epidemiological year in a high-endemicity area. The probability of severe and lethal clinical outcomes was higher during 2020 and 2021.

## INTRODUCTION

Dengue is the most significant viral hemorrhagic disease and a vector-borne virus (transmitted by arthropods) with widespread distribution in tropical and subtropical regions worldwide.^1^^–^^3^ Between 50 and 100 million infections occur yearly, primarily in the Americas, South and Southeast Asia, and the Western Pacific, with circulation in Africa and recent outbreaks in the United States and parts of Europe.^4^^–^^6^ In Latin America, more than 4 million people are infected with dengue each year, with an increased number of cases over the last 5 years.^7^^,^^8^ The countries with reported outbreaks in the Americas include Bolivia, Brazil, Colombia, the Dominican Republic, Guatemala, Honduras, Nicaragua, Mexico, Peru, Puerto Rico, and Venezuela.^1^ In this region, dengue cases have increased significantly over the past four decades, from 1.5 million cases between 1980 and 1989 to 17.5 million between 2010 and 2019. According to WHO reports, until 2023, the highest number of dengue cases recorded to date was in 2019, with more than 3.18 million cases, 28,208 severe cases, and 1,823 deaths.^6^ In 2023, 4,594,823 dengue cases were reported, 44.7% of which were laboratory-confirmed. There were 9,198 cases of severe dengue and 2,467 deaths attributed to the disease. By 2024, 12,994,447 dengue cases had been reported, of which 6,588,863 had been confirmed. In addition, 22,665 cases of severe dengue and 8,151 deaths have been recorded.^9^

Colombia has one of the highest mortality rates in the subregion (0.1%), in addition to accelerated transmission of the virus through epidemic cycles every 2 or 3 years, an increase in outbreaks of severe dengue, simultaneous circulation of different serotypes, and vector circulation in almost 90% of the national territory.^4^^,^^10^ In 2023, 143,136 probable dengue cases were reported (64.9% of which were laboratory-confirmed), along with 1,358 cases of severe dengue and 67 deaths attributed to dengue.^9^ In 2024, according to the Pan American Health Organization, Colombia reported 290,072 dengue cases, 2,697 severe dengue cases, and 182 deaths.^9^ By municipality of origin, Cali has registered the highest number of cases reported per epidemiological year at the national level. In 2023, 131,784 cases were reported, with incidence rates exceeding 500 per 100,000 inhabitants.^9^^,^^11^

Dengue virus (DENV) infection presents with a broad spectrum of clinical manifestations.^5^^,^^12^^–^^17^ Associated factors, such as age (higher frequency of vascular leak and shock in children), sex (mainly men), and some comorbidities (particularly asthma, obesity, diabetes, sickle cell disease, and cardiovascular disease), pregnancy, nutritional status, immunocompromised conditions, serotype, previous dengue infections, and the quality of health care, have been identified in patients with severe dengue.^5^^,^^12^^–^^17^ Similarly, periods of endemicity or population-level transmission have been linked to a threefold increase in dengue mortality rates every 10 years in Central and South America.^18^^–^^20^

Year after year, changes have been observed in the clinical course of patients due to temporal heterogeneities related to the existence of genomic variants of the virus and high vector densities,^18^^,^^19^ among other factors, such as the increase and subsequent decrease in antibodies in individuals after the Zika and dengue epidemics.^21^ Although there is information on the clinical course of dengue to date, it has yet to be addressed in terms of outcomes and the probability of occurrence by degree of severity and periods of endemicity.^22^^–^^24^ Therefore, the aim for the present study is to determine the characteristics of the clinical course and the differences in severe and fatal outcomes by degree of endemicity in patients with dengue treated in healthcare institutions in Santiago de Cali, Colombia, between 2019 and 2022.

For the purposes of the present study, endemicity was conceptualized as the level or intensity at which dengue remains consistently present within a given population. Epidemic periods were characterized by a sudden increase in the number of cases.

## MATERIALS AND METHODS

### Study design.

A historical, observational, longitudinal study with a single cohort was conducted, and the follow-up period extended from the day of the patient’s consultation or admission to the healthcare institution (*t* = 0) to either 10 days later or until discharge, whether the patient was alive or deceased.

The current study spanned 4 epidemiological years, establishing distinct scenarios on the basis of data provided by Cali’s District Health Secretariat: two epidemic dengue periods (2019–2020), during which the number of observed cases exceeded expected levels, with increases of 44.3% and 69.6% compared with the previous year, and two potential endemic periods (2021–2022), marked by sustained transmission with moderate or low incidence, in which case numbers declined by 12.7% and 3.8%, respectively, relative to the preceding year.

### Target population.

Pediatric and adult patients with a probable or confirmed diagnosis of dengue who were treated at three different health institutions: a first-level public network health institution offering primary care, and two third-level health institutions providing complementary and specialized care (both public and private).

### Study population.

Cases with a discharge diagnosis of dengue, confirmed by the treating physician through clinical evaluation or available laboratory tests, with no age limit, were treated in three health institutions in Santiago de Cali, Colombia. Selected patients met the definitions of a probable dengue case (a patient from an endemic area who meets the definition of dengue with or without warning signs), a probable severe dengue case (any dengue case [severe] that involves severe plasma extravasation, severe hemorrhages, or serious organ damage), or a confirmed dengue case with serological IgM or virological tests, such as viral isolation or reverse-transcriptase polymerase chain reaction, according to the guidelines of the Ministry of Health and Social Protection of Colombia.^4^ Suspected cases confirmed as negative were excluded according to the adjustments reported in the notification form of the National Public Health Surveillance System (SIVIGILA; adjustments 6: Discard and D: Typing error).

### Sample and sampling strategy.

A sample size of 1,038 medical records was obtained by taking the highest estimated value among the different outcomes considered (admission to the hospital, admission to the intensive care unit [ICU], days of hospital stay, and mortality). The sample size was calculated for each institution using the proportional stratified sampling formula, with an estimated proportion and mean,^25^ on the basis of data obtained from the Cali Public Health Department report for 2019 to 2022. The strata correspond to each year of study. A confidence level of 95% and an estimation error of 0.05 were established.

The cases were selected from the total number of patients notified to SIVIGILA of the National Institutes of Health of Colombia (INS), specifically those with the following data sheets completed: 210 Dengue, 220 Severe Dengue, and 580 Dengue Mortality. The selection was conducted using simple random sampling (random numbers) by year of notification and institution until the required sample size was reached. Selected patients with limitations in accessing information or with inconsistencies related to the identification number were replaced randomly, respecting the established sampling criteria.

### Data collection.

Before the implementation phase, a capture format was designed to collect information on the Azure Static platform (Microsoft Corp., Redmond, WA) using PostgreSQL (PostgreSQL Global Development Group, Santa Barbara, CA). The medical personnel responsible for data collection were trained on the clinical aspects of dengue, the operational characteristics of serological tests, the operational definitions of the variables, and the format for capturing and uploading information. The information was obtained from the patients’ medical records, institutional records (laboratory tests), and SIVIGILA notification forms.

Among the variables of interest is the healthcare regimen, which corresponds to the patient’s health insurance type. The subsidized health insurance system in Colombia is designed to provide coverage to low-income individuals and families who cannot afford health insurance. This system is funded by the government and is designed to ensure that all citizens have access to essential health services, regardless of their financial situation. The contributory health insurance system is intended for individuals with the financial means to contribute to their health insurance. This system is funded through contributions from employees, employers, and self-employed individuals based on a percentage of the individual’s income. It ensures that those who can afford to pay for health insurance contribute to the overall healthcare system.

The variable duration of incapacity refers to the number of days of home rest and outpatient management authorized by the attending physician. It applies to adults, school-aged children, and early childhood children who require formal support to justify absence from work, school, or early childhood care centers such as nurseries or Child Development Centers (CDCs). In Colombia, both nurseries and CDCs require official medical documentation to justify a child’s absence. This support is essential for maintaining the assigned enrollment slot and ensuring continuity of care.

After completing the data collection phase, the cases were classified according to the INS criteria^4^ as follows: 1) dengue without warning signs, 2) dengue with warning signs, and 3) severe dengue (Supplemental Table 1).

## STATISTICAL ANALYSES

In the descriptive analysis, the quantitative variables were summarized using measures of central tendency (mean, median) and dispersion (SD, interquartile range [IQR]) according to their distributions. Similarly, measures of absolute and relative frequencies were established for the qualitative variables. In addition, the mortality rate, the cumulative incidence of hospitalization and ICU admission, and the number of days of incapacity, hospitalization, and ICU stay were estimated.

Subgroup analyses were performed by comorbidity variables, patients’ sociodemographic characteristics, dengue type, and date of notification. Statistical differences were evaluated using the nonparametric χ^2^ test or Fisher’s exact test for categorical variables and the nonparametric Kruskal–Wallis test for quantitative variables, as appropriate; 95% CIs were calculated for the cumulative incidence of hospitalization, ICU admission, and mortality.

To identify variables associated with dengue with warning signs and severe dengue (base conditions and sociodemographic and clinical characteristics of patients), a logistic regression model was estimated using a non-conditional multinomial link, and odds ratios were calculated relative to the reference categories. The operational characteristics of the model were assessed to evaluate its performance (sensitivity, specificity, predictive values, and F1 score).^26^ In the variable employment-based health coverage, four records were omitted (one patient affiliated with the special regimen and three patients who lacked reporting) because this frequency imbalance interfered with estimating the model coefficients.

For the survival analysis, the estimation of the differences in the variables of admission to the hospital, days of hospitalization, and mortality within 10 days of follow-up or until the patient’s discharge (counted from the date of admission to the health institution with a diagnosis of dengue) was conducted using the competing risks method. This was due to the difficulty of estimating the risk function using conventional Kaplan–Meier curves or Cox regression.^27^ Therefore, the cumulative incidence curve was calculated for the variables of interest, the proportional hazards assumption was validated, and the incidence curves or the conditional probability of the event occurring in the next instant of time (given that it has not happened before) were compared using the hazard ratio.

## RESULTS

### Sociodemographic characteristics, clinical manifestations, comorbidities, and therapeutic management.

A total of 1,038 dengue cases were included in the present study: 473 (45.5%) from a private third-level institution, 316 (30.4%) from a public third-level institution, and 250 (24%) from a public first-level institution in Cali, Colombia. The median age of patients with severe dengue was 10 years (Quartile [Q]1–Q3: 5–20 years), which was younger than that of patients with non-severe dengue cases (22 years; Q1–Q3: 11–39 years; *P* <0.01). The median age of hospitalized patients was 12 years (Q1–Q3: 6–22 years), and for non-hospitalized patients, it was 19 years (Q1–Q3: 10–35 years). Approximately 52% of the patients were women. According to institution type, severe dengue cases predominated in the high-complexity public facility (75.9%). In contrast, the low-complexity public facility exhibited an intermediate distribution of cases with and without warning signs (61.6% and 34.8%), with a low proportion of severe dengue (3.6%). This distribution reveals marked differences between groups, with a *P*-value of <0.001 indicating a statistically significant association between institution type and participant group. The patients had employment-based health insurance coverage (48%), and most patients with severe dengue were in the subsidized program. More than 70% of patients lived in urban areas (Table 1).

**Table 1:** Sociodemographic, comorbidity, and initial contact characteristics of patients with dengue by level of severity, Santiago de Cali, 2019–2022

Variables, *n* (%)	DWWS, *n* = 277	DWS, *n* = 487	Severe Dengue, *n* = 274	Total, *N* = 1,038	*P*-Value
Age					<0.01
Median (Q1-Q3)	22 (11–39)	14 (8–26.50)	10 (5–20)	14 (8–28)	
Sex					0.73
Female	143 (51.62)	251 (51.54)	149 (54.38)	543 (52.31)	
Institution					<0.01
Third-level private	166 (59.93)	281 (57.70)	26 (9.49)	473 (45.57)	
Third-level public	24 (8.66)	52 (10.68)	239 (87.23)	315 (30.35)	
First-level public	87 (31.41)	154 (31.62)	9 (3.28)	250 (24.08)	
Regimen					<0.01
Employment-based health coverage	167 (60.29)	282 (57.91)	48 (17.52)	497 (47.88)	
Uninsured	22 (7.94)	40 (8.21)	39 (14.23)	101 (9.73)	
Subsidized*	88 (31.77)	164 (33.68)	184 (67.15)	436 (42)	
Area of residence					0.31
Urban Cali	212 (76.53)	375 (77)	146 (53.28)	733 (70.62)	
Rural Cali	16 (5.78)	24 (4.93)	5 (1.82)	45 (4.34)	
Outside Cali (attended in Cali)	1 (0.36)	4 (0.82)	0 (0)	5 (0.48)	
Does not report	48 (17.33)	84 (17.25)	123 (44.89)	255 (24.57)	
Comorbidity					
Diabetes mellitus	9 (3.25)	5 (1.03)	5 (1.82)	19 (1.83)	<0.01
Arterial hypertension	21 (7.58)	25 (5.13)	19 (6.93)	65 (6.26)	<0.01
Chronic hematological	5 (1.81)	7 (1.44)	11 (4.01)	23 (2.22)	<0.01
Chronic kidney disease	2 (0.72)	3 (0.62)	7 (2.55)	12 (1.16)	<0.01
Severe cardiovascular	4 (1.44)	12 (2.46)	3 (1.09)	19 (1.83)	0.06
Peptic ulcer disease	6 (2.17)	13 (2.67)​	2 (0.73)	21 (2.02)	0.32
Autoimmune diseases	3 (1.08)	7 (1.44)	2 (0.73)	12 (1.16)	0.14
Epilepsy	3 (1.08)	8 (1.64)	2 (0.73)	13 (1.25)	0.13
COPD	2 (0.72)	2 (0.41)	2 (0.73)	6 (0.58)	0.01
COVID-19	5 (1.81)	11 (2.26)	4 (1.46)	20 (1.93)	0.01
Coinfections	17 (6.14)	3 7 (7.60)	34 (12.41)	88 (8.48)	0.02
Female participants	*n* = 143	*n* = 251	*n* = 149	*n* = 543	0.09
Pregnant women	11 (7.69)	13 (5.17)	15 (10.06)	39 (7.18)	
Consultation opportunity (days)					–
Median (Q1–Q3)	4 (2–5)	4 (3–5)	4 (3–6)	4 (2–5)	
Minimum	0	0	3	0	
Maximum	15	26	30	30	
Hospital readmission	29 (10.47)	38 (7.80)	6 (2.19)	73 (7.03)	0.15
Median (Q1–Q3)	0 (0-0)	0 (0-0)	0 (0-0)	0 (0-0)	
Minimum	0	0	0	0	
Maximum	3	4	1	4	
Number of readmissions					<0.01
0	248 (89.53)	449 (92.20)	268 (97.81)	965 (92.97)	
1	24 (8.66)	31 (6.37)	6 (2.19)	61 (5.88)	
2	3 (1.08)	4 (0.82)	0 (0)	7 (0.67)	
3	2 (0.72)	1 (0.21)	0 (0)	3 (0.29)	
4	0 (0)	2 (0.41)	0 (0)	2 (0.19)	
Referred from another institution	22 (7.94)	61 (12.53)	159 (58.03)	242 (23.31)	<0.01

COPD = chronic obstructive pulmonary disease; COVID-19 = coronavirus disease 2019; DWS = dengue with warning signs; DWWS = dengue without warning signs; Q = quartile.

* The subsidized health insurance system in Colombia is designed to provide healthcare coverage to low-income individuals and families who cannot afford to pay for health insurance. This system is funded by the government and is designed to ensure that all citizens have access to essential health services, regardless of their financial situation.

The median timeline of patient consultation after symptom onset was 4 days (Q1–Q3: 2–5 days). Seventy-five percent of patients with severe dengue presented to the emergency department within 6 days of symptom onset, whereas patients with warning signs sought care within 5 days of symptom onset. Approximately 7% of patients had between one and four readmissions; 58% of patients with severe dengue were referred from another health institution (Table 1).

Among participants with chronic hematological diseases (2%; *P* <0.01) and chronic kidney disease (1.2%; *P* <0.01; *P* = 0.02), severe dengue was reported more frequently. Dengue with warning signs was more common among patients with high blood pressure (*P* <0.01), severe cardiovascular disease (*P* = 0.06), and coinfections (*P* = 0.02; Table 1). High blood pressure (*P* = 0.09) and chronic kidney disease (*P* = 0.09) were associated with severe dengue. In contrast, peptic ulcer disease (*P* = 0.01) and readmission to the health institution (*P* = 0.05) decreased the probability of developing severe dengue (Supplemental Tables 2 and 3). Hematological conditions (*n* = 7; *P* <0.01), coinfections (*n* = 18; *P* <0.01), and pregnancy (*n* = 15; *P* <0.01) exhibited significant differences regarding admission to the ICU.

Patients with warning signs more frequently presented with abdominal pain (45.8%), vomiting (40.7%), diarrhea (33.9%), rash (29.4%), epistaxis (8.21%), petechiae (4.7%), hematuria (3.5%), and gingival bleeding (3.5%; *P* <0.001). Cases with liver involvement (91.2%), hepatomegaly (18.25%), renal failure (9.5%), altered state of consciousness (6.6%), and loss of muscle sensitivity or strength (1.4%) were primarily classified as severe dengue (*P* <0.01; Table 2). More than 84% of the patients had fever, and leukocyte (*P* <0.01) and platelet counts (*P* <0.01) were higher in patients with severe dengue (Table 2).

**Table 2:** Signs and initial symptoms of patients with dengue by level of severity, Santiago de Cali, 2019–2022

Variables, *n* (%)	DWWS, *n* = 277	DWS, *n* = 487	Severe Dengue, *n* = 274	Total, *N* = 1,038	*P*-Value
Hyporexia	22 (7.94)	59 (12.11)	53 (19.34)	134 (12.91)	<0.01
Arthralgias	69 (24.91)	96 (19.70)	61 (22.26)	226 (21.77)	<0.01
Myalgias	86 (31.05)	116 (23.82)	76 (27.74)	278 (26.78)	<0.01
Headache	126 (45.49)	186 (38.19)	85 (31.02)	397 (38.25)	<0.01
Nausea	20 (7.22)	80 (16.43)	20 (7.30)	120 (11.56)	<0.01
Vomiting	0 (0)	198 (40.66)	120 (43.80)	318 (30.64)	<0.01
Diarrhea	0 (0)	165 (33.88)	66 (24.09)	231 (22.25)	<0.01
Rash	78 (28.16)	143 (29.36)	45 (16.42)	266 (25.63)	<0.01
Hematuria	0 (0)	17 (3.49)	6 (2.19)	23 (2.22)	<0.01
Petechiae	0 (0)	23 (4.72)	23 (8.39)	46 (4.43)	<0.01
Epistaxis	0 (0)	40 (8.21)	41 (14.96)	81 (7.80)	<0.01
Gingival bleeding	0 (0)	17 (3.49)	7 (2.55)	24 (2.31)	<0.01
Abdominal pain	0 (0)	223 (45.79)	139 (50.73)	362 (34.87)	<0.01
Alteration of the state of consciousness	0 (0)	7 (1.44)	18 (6.57)	5 (2.41)	<0.01
Loss of muscle sensation/strength	0 (0)	0 (0)	4 (1.46)	4 (0.39)	<0.01
Target organ involvement	0 (0)	0 (0)	253 (92.34)	253 (24.37)	<0.01
Hepatomegaly	0 (0)	4 (0.82)	50 (18.25)	54 (5.20)	<0.01
Renal failure	0 (0)	0 (0)	26 (9.49)	26 (2.50)	<0.01
Liver involvement	0 (0)	0 (0)	250 (91.24)	250 (24.08)	<0.01
Female participants	(*n* = 143)	(*n* = 251)	(*n* = 149)	(*n* = 543)	<0.01
Women with vaginal bleeding	0 (0)	6 (2.39)	6 (4.02)	12 (2.20)	
Fever	208 (75.09)	423 (86.86)	242 (88.32)	873 (84.10)	0.02
SRD	9 (3.25)	23 (4.72)	11 (4.01)	43 (4.14)	0.19
RR median (Q1–Q3)	19 (18–20)	20 (18–22)	20 (18–24)	20 (18–22)	<0.01
HR median (Q1–Q3)	98 (82–114)	101 (85–119)	98 (82–120)	100 (83–116)	0.02
SBP median (Q1–Q3)	113 (103–127)	108 (99–119)	110 (98–129)	110 (100–122.8)	<0.01
DBP median (Q1-Q3)	74 (66–82)	68 (60–76)	70 (60–80)	70 (61–79)	<0.01
Leukocyte count (µl), median (Q1–Q3)	4,300 (3,200–7,470)	3,940 (2,730–6,710)	5,935 (3,405–9,858)	4,405 (3,002–7,530)	<0.01
Platelet count (mcL), median (Q1–Q3)	90,000 (16,800–147,000)	81,000 (15,500–161,000)	110,500 (47,000–180,000)	91,000 (22,125–167,750)	<0.01
Hematocrit (%), median (Q1–Q3)	41.60 (38.40–45.40)	40.60 (37.30–43.80)	39 (34.30–43.10)	40.60 (36.8–44.00)	<0.01
Hemoglobin (g/dL), median (Q1–Q3)	14.10 (13.00–15.40)	13.90 (12.70–15.00)	13 (11.30–14.60)	13.70 (12.50–15.00)	<0.01

DBP = diastolic blood pressure on admission; DWS = dengue with warning signs; DWWS = dengue without warning signs; HR = heart rate upon admission; Q = quartile; RR = respiratory rate on admission; SBP = systolic blood pressure on admission; SRD = signs of respiratory distress upon admission.

More than 65% of patients received acetaminophen, and 36% received nonsteroidal anti-inflammatory drugs. Approximately 74% and 84.1% of patients with dengue without warning signs had home management and outpatient follow-up, respectively. A total of 67.3% of hospitalized patients received intravenous therapy with crystalloids, and 16.9% received a second infusion scheme. All patients in the ICU received initial intravenous therapy. The highest proportions of cases in which colloids and second fluid boluses were administered were among patients with severe dengue (88% and 66.7%, respectively). Among patients who received blood transfusions, the majority had warning signs (66.7%).

### Mortality, hospitalization, and ICU admission by dengue severity level and epidemiological year.

Of the total number of patients, 17 died: six from multiple organ failure, five from acute myocardial infarction, two from cerebral herniation, two from an unestablished cause, one from intracranial hemorrhage, and one from septic shock. Of those who died, 88.2% had severe dengue, and 11.8% had dengue with warning signs. The median age of the patients was 37.50 years (Q1–Q3: 17–63 years); 25% were under 17 years of age, and 11.8% were under 5 years of age. A total of 58.8% were male and belonged to the subsidized health insurance program (*n* = 12; *P* <0.01); six had coinfections, five had hypertension, and three had type 2 diabetes mellitus.

A higher mortality rate was estimated in patients with severe dengue (5.47) compared with those with and without warning signs (0.41 and 0, respectively). More than 50% of patients with warning signs and severe dengue were admitted to the hospital, compared with those without warning signs (*P* <0.01). Patients with severe dengue were more frequently admitted to the ICU (20%) compared with those with and without warning signs (*P* <0.01). The incidence of hospitalization in the study population was 56 cases per 100 patients during the follow-up period. When analyzed by severity subgroups, hospitalization incidence was 66 cases per 100 patients with severe dengue and 87 cases per 100 patients with warning signs. A cumulative incidence of ICU admission of nine cases per 100 patients was observed; the highest incidence was estimated among patients with severe dengue (20 cases per 100 patients; Table 3). By epidemiological year, case fatality was higher in 2020 and 2021, reaching values of 2.08. Between 2019 and 2022, the incidence of hospitalization was ∼37 cases per 100 patients, with a higher incidence in 2019 and 2020 (64 and 53 cases per 100 patients, respectively). The highest incidence of ICU admission occurred in 2022 (15 cases per 100 patients; Table 4).

**Table 3:** Outcomes of interest and cumulative incidences by level of dengue severity, Santiago de Cali, 2019–2022

Variables, *n* (%)	DWWS, *n* = 277	DWS, *n* = 487	Severe Dengue, *n* = 274	Total, *N* = 1,038	*P*-Value
Final condition					<0.01
Alive	277 (100)	485 (99.59)	259 (94.53)	1,021 (98.36)	
Dead	0 (0)	2 (0.41)	15 (5.47)	17 (1.64)	
Hospitalization					<0.01
Yes	183 (66.06)	255 (52.36)	238 (86.86)	587 (56.54)	
No	94 (33.93)	232 (47.63)	36 (13.13)	451 (43.44)	
ICU admission					<0.01
Yes	5 (1.81)	32 (6.57)	56 (20.44)	93 (8.96)	
No	272 (98.19)	455 (93.43)	218 (79.56)	945 (91.04)	
Incidence of hospitalization*	66.06	52.36	86.86	56.54	–
Incidence of ICU admission*	1.81	6.57	20.44	8.96	–
Mortality	0	0.41​	5.47	1.64	–

DWS = dengue with warning signs; DWWS = dengue without warning signs; ICU = intensive care unit.

* Study population at risk (multiplication coefficient: 100).

**Table 4:** Outcomes of interest and accumulated incidences by epidemiological year, Santiago de Cali, 2019–2022

Variables, *n* (%)	2019, *n* = 200	2020, *n* = 527	2021, *n* = 192	2022, *n* = 119	*P*-Value
Final condition					0.12
Alive	200 (100)	516 (97.91)	188 (97.92)	117 (98.32)	
Dead	0 (0)	11 (2.09)	4 (2.08)	2 (1.68)	
Hospitalization					0.04
Yes	128 (64)	279 (52.94)	107 (44.27)	73 (38.66)	
No	72 (36)	248 (47.06)	85 (55.73)	46 (61.34)	
ICU admission					0.08
Yes	16 (8)	41 (7.78)	18 (9.38)	18 (15.13)	
No	184 (92)	486 (92.22)	174 (90.62)	101 (84.87)	
Incidence of hospitalization*	64	52.94	44.27	36.66	–
Incidence of ICU admission*	8	7.78	9.38	15.13	–
Mortality*	0	2.08	2.08	1.68	–

ICU = intensive care unit.

* Population at risk of the study (multiplication coefficient: 100).

Across the three institutions evaluated, all patients diagnosed with dengue without warning signs and dengue with warning signs survived; only two deaths were reported among patients with dengue with warning signs. Twelve deaths were recorded in cases of severe dengue, all of which occurred in the public tertiary-level institution. Regarding hospitalization, patterns differed by institution: at the public primary-level institution, most admissions involved patients with warning signs (43.5%). In the public tertiary-level institution, hospitalization predominantly occurred in patients with severe dengue (88.7%). In the private tertiary-level institution, admissions were concentrated among patients with warning signs (53.4%). Regarding ICU admissions, both public and private tertiary-level institutions primarily admitted patients with severe dengue and those with warning signs to the ICU. The highest frequency of ICU admission for severe dengue occurred in the public institution (20.5%), whereas the private institution reported more cases of ICU admission for patients with warning signs (9.3%). The incidence of hospitalization was highest among patients with severe dengue across all three institutions, at 77.8%, 88.7%, and 73.1%, respectively. Similarly, ICU admission was more frequent among patients with severe dengue treated in tertiary-level institutions (Supplemental Table 5).

### Duration of incapacity, hospitalization, and ICU stay by dengue severity level and epidemiological year.

Patients with warning signs experienced incapacity for up to 90 days, whereas those without warning signs experienced up to 17 days of incapacity. Patients diagnosed with severe dengue experienced incapacity for up to 15 days. The average hospital stay was estimated at 2.0 days, and the average ICU stay was estimated at 4.5 days. Prolonged hospitalizations were primarily observed among patients with warning signs, with a median of 3 days (range: 0–25 days). Patients with severe dengue experienced hospital stays of up to 53 days, whereas those with warning signs experienced stays of up to 25 days. Regarding ICU stays, the longest durations were observed in patients with severe dengue and those with warning signs (up to 24 and 14 days, respectively; Table 5). The longest hospital and ICU stays were recorded among patients treated in 2020, with maximum values of 63 and 21 days, respectively (Table 6).

**Table 5:** Duration of incapacity, days of hospitalization, and intensive care unit stay of patients with dengue by level of severity, Santiago de Cali, 2019–2022

Variables, *n* (%)	DWWS, *n* = 277	DWS, *n* = 487	Severe Dengue, *n* = 274	Total, *N* = 1,038
Duration of incapacity				
Median (Q1–Q3)	0 (0-0)	0 (0-0)	0 (0-0)	0 (0-0)
Minimum	0	0	0	0
Maximum	17	90	15​	90
Hospital stay				
Median (Q1–Q3)	2.5 (1–4)	3 (2–4)	2 (2–4)	2 (2–4)
Minimum	0	0	1	0
Maximum	63	25	53	63
ICU stay				
Mean (SD)	3 (±1.41)	4.33 (±2.94)	4.70 (±4.46)	4.48 (±3.88)
Minimum	2	1	0	0
Maximum	5	14	24	24

DWS = dengue with warning signs; DWWS = dengue without warning signs; ICU = intensive care unit; Q = quartile.

**Table 6:** Duration of incapacity, days of hospitalization, and intensive care unit stay of patients with dengue by epidemiological year, Santiago de Cali, 2019–2022

Variables	2019, *n* = 200	2020, *n* = 527	2021, *n* = 192	2022, *n* = 119
Duration of incapacity				
Median (Q1–Q3)	2 (1–3)	2 (1–4)	3 (2–5)	3 (2–6)
Minimum	0	0	0	0
Maximum	5	18	10	90
Hospital stay				
Median (Q1–Q3)	2 (1–3)	2 (1–4)	3 (2–5)	3 (2–6)
Minimum	0	0	0	1
Maximum	21	63	32	21
ICU stay				
Median (Q1–Q3)	3 (2–4)	4 (2–5)	4 (2–7.25)	3 (2.75–5.75)
Minimum	1	0	1	1
Maximum	14	21	14	24

ICU = intensive care unit; Q = quartile.

### Differences in dengue hospital admissions, hospitalization days, and mortality between epidemic and endemic periods.

**Figure 1.**
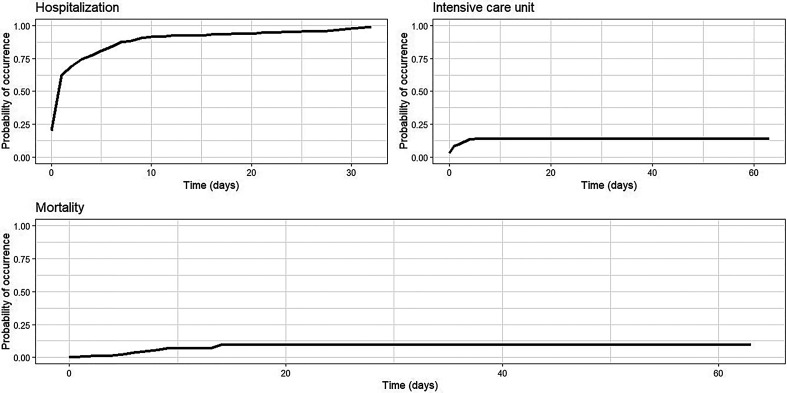
Risk function for hospitalization, intensive care unit admission, and mortality of patients with dengue, Santiago de Cali, 2019–2022.

The cumulative incidence of hospitalization increased rapidly within the first 10 days after initial healthcare contact, reaching 91.4% (95% CI: 86.9–94.4%). This trend is supported by the hospitalization curve, which shows a steep ascent during the early days and plateaus at ∼1.0 by day 10, remaining stable through day 30. From day 15 onward, the rate stabilized above 92.2%, with no further increase observed between days 25 and 30, suggesting saturation of the event and early resolution of hospitalization needs (Tables 7–9, Figure 1, and Supplemental Table 6).

**Table 7:** Cumulative incidence of hospitalization of patients with dengue, Santiago de Cali, 2019–2022

Hospitalization: Cumulative Incidence (95% CI)						
0 days	5 days	10 days	15 days	20 days	25 days	30 days
0.201 (0.177–0.226)	0.808 (0.767–0.842)	0.914 (0.869–0.944)	0.922 (0.876–0.951)	0.939 (0.891–0.967)	0.957 (0.906–0.980)	0.978 (0.916–0.995)

**Table 8:** Cumulative incidence of intensive care unit admission of patients with dengue, Santiago de Cali, 2019–2022

ICU Admission: Cumulative Incidence (95% CI)						
0 days	10 days	20 days	30 days	40 days	50 days	60 days
0.0289 (0.019–0.0404)	0.141 (0.112–0.173)	0.141 (0.112–0.173)	0.141 (0.112–0.173)	0.141 (0.112–0.173)	0.141 (0.112–0.173)	0.141 (0.112–0.173)

ICU = intensive care unit.

**Table 9:** Cumulative incidence of mortality of patients with dengue, Santiago de Cali, 2019–2022

Mortality: Cumulative Incidence (95% CI)						
0 days	10 days	20 days	30 days	40 days	50 days	60 days
0.001 (0.000–0.005)	0.068 (0.034–0.117)	0.095 (0.042–0.175)	0.095 (0.042–0.175)	0.095 (0.042–0.175)	0.095 (0.042–0.175)	0.095 (0.042–0.175)

In contrast, ICU admission exhibited an early plateau, with a cumulative incidence of 14.1% (95% CI: 11.2–17.3%) by day 5, which remained unchanged through day 60. The corresponding graph reveals a flat trajectory near zero throughout the entire follow-up period, indicating that the risk of requiring intensive care was concentrated in the initial phase and did not increase over time. This pattern suggests that clinical deterioration requiring ICU support is both infrequent and temporally constrained (Tables 7–9, Figure 1, and Supplemental Table 6).

The mortality rate exhibited a gradual rise, beginning at 0% (95% CI: 0–0.1%) on day 0 and reaching 9.5% (95% CI: 4.2–17.5%) by day 20. The mortality curve reflects this progression, with a slow but steady increase over time, a slight uptick around day 20, and continued elevation through day 60. The rate remained stable thereafter, suggesting that most fatal outcomes occurred within the first 3 weeks. The wider CIs and the gentle slope of the mortality graph indicate greater uncertainty and variability in mortality estimates than for hospitalization and ICU admission (Tables 7–9, Figure 1, and Supplemental Table 6).

**Figure 2.**
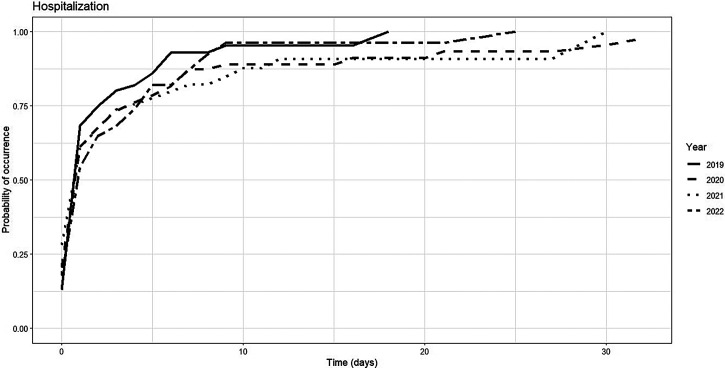
Risk function for hospitalization in dengue patients by epidemiological year, Santiago de Cali, 2019–2022.

The cumulative incidence of hospitalization increased rapidly across all years, with notable differences in the timing of onset and stabilization. In 2019, hospitalization reached 85.9% (95% CI: 75.7–92.1%) by day 5 and stabilized at 95.3% from day 10 onward. In contrast, the years 2020, 2021, and 2022 exhibited higher initial rates (17.6–28.1% on day 0), rising to 81.9% by day 5 and 96.1% by day 10, with no change through day 30. The corresponding probability curves reflect steeper increases in 2019 and 2020, whereas 2021 and 2022 exhibit more gradual trajectories, reaching near-maximal values between days 20 and 25. These patterns suggest earlier clinical severity and hospitalization saturation in pandemic years (Tables 10–12, Figure 2, and Supplemental Table 7).

**Table 10:** Cumulative incidence of hospitalization of patients with dengue by epidemiological year, Santiago de Cali, 2019–2022

Hospitalization: Cumulative Incidence (95% CI) Per Year							
Year	0 days	5 days	10 days	15 days	20 days	25 days	30 days
2019	0.130 (0.088–0.181)	0.859 (0.757–0.921)	0.953 (0.813–0.989)	0.953 (0.813–0.989)	–	–	–
2020	0.205 (0.172–0.240)	0.784 (0.721–0.835)	0.889 (0.806–0.938)	0.889 (0.806–0.938)	0.911 (0.815–0.959)	0.933 (0.824–0.976)	0.956 (0.827–0.989)
2021	0.281 (0.219–0.346)	0.776 (0.665–0.854)	0.877 (0.736–0.945)	0.908 (0.752–0.968)	0.908 (0.752–0.968)	0.908 (0.752–0.968)	1.0 (1.0-1.0)
2022	0.176 (0.114–0.250)	0.819 (0.690–0.898)	0.961 (0.676–0.996)	0.961 (0.676–0.996)	0.961 (0.676–0.996)	1.0 (1.0–1.0)	–

**Table 11:** Cumulative incidence of intensive care unit admission of patients with dengue by epidemiological year, Santiago de Cali, 2019–2022

ICU: Cumulative Incidence (95% CI) Per Year							
Year	0 days	10 days	20 days	30 days	40 days	50 days	60 days
2019	0.030 (0.012–0.061)	0.129 (0.069–0.208)	0.129 (0.069–0.208)	–	–	–	–
2020	0.036 (0.022–0.054)	0.110 (0.078–0.147)	0.110 (0.078–0.147)	0.110 (0.078–0.147)	0.110 (0.078–0.147)	0.110 (0.078–0.147)	0.110 (0.078–0.147)
2021	0.016 (0.004–0.042)	0.159 (0.094–0.241)	0.159 (0.094–0.241)	0.159 (0.094–0.241)	–	–	–
2022	0.017 (0.003–0.054)	0.235 (0.139–0.346)	0.235 (0.139–0.346)	0.235 (0.139–0.346)	0.235 (0.139–0.346)	0.235 (0.139–0.346)	–

ICU = intensive care unit.

**Table 12:** Cumulative incidence of mortality of patients with dengue by epidemiological year, Santiago de Cali, 2019–2022

Mortality: Cumulative Incidence (95% CI) Per Year							
Year	0 days	10 days	20 days	30 days	40 days	50 days	60 days
2019	0	0	0	0	–	–	–
2020	0.002 (0.000–0.010)	0.112 (0.040–0.225)	0.112 (0.040–0.225)	0.112 (0.040–0.225)	0.112 (0.040–0.225)	0.112 (0.040–0.225)	0.112 (0.040–0.225)
2021	0	0.088 (0.019–0.223)	–	0.189 (0.035–0.435)	–	–	–
2022	0	0.026 (0.005–0.082)	0.026 (0.005–0.082)	0.026 (0.005–0.082)	0.026 (0.005–0.082)	0.026 (0.005–0.082)	–

**Figure 3.**
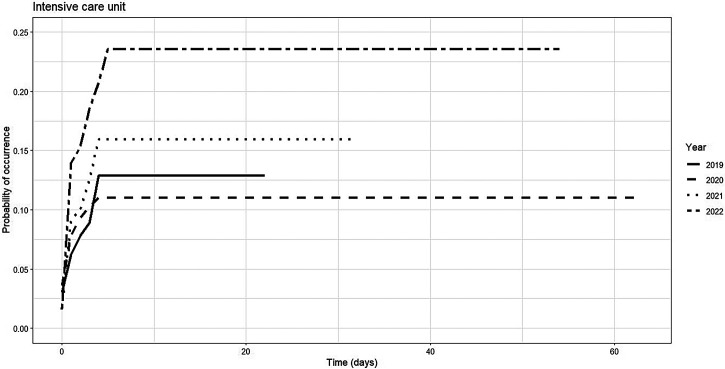
Risk function for intensive care unit admission in dengue patients by epidemiological year, Santiago de Cali, 2019–2022.

The cumulative incidence of ICU admission varied significantly between 2019 and 2022, both in magnitude and temporal progression. In 2019, the incidence was 3.0% (95% CI: 1.2–6.1%) on day 0, increasing to 12.9% (95% CI: 6.9–20.8%) by day 10. No data were reported beyond this point, suggesting an early peak in ICU admissions that year. In 2020, the incidence reached 11.0% (95% CI: 7.8–14.7%) by day 10 and remained constant through day 60. This pattern indicates an early stabilization of ICU admission risk. In 2021, a lower initial incidence was observed (1.6%; 95% CI: 0.4–4.2%), followed by a more pronounced increase by day 10, reaching 15.9% (95% CI: 9.4–24.1%), with no further changes through day 30. In 2022, the incidence was 1.7% (95% CI: 0.3–5.4%) on day 0, rising rapidly to 23.5% (95% CI: 13.9–34.6%) by day 10 and remaining stable through day 60. Graphically, the 2019 curve exhibits a delayed rise, whereas the 2020 curve reveals an earlier and lower plateau. The 2021 and 2022 curves exhibit steeper increases and higher final values, particularly in 2022. These patterns suggest a shift toward earlier and more frequent ICU admissions in pandemic years, potentially influenced by evolving clinical criteria, hospital capacity, or disease severity (Tables 10–12, Figure 3, and Supplemental Table 7).

**Figure 4.**
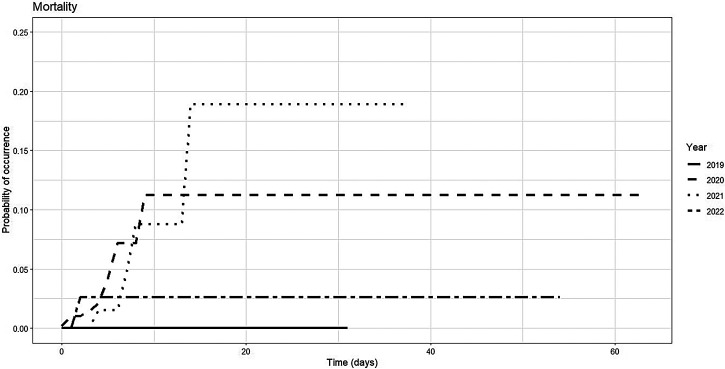
Risk function of mortality in dengue patients by epidemiological year, Santiago de Cali, 2019–2022.

Regarding mortality, no events were recorded in 2019 until day 30. In 2020, the cumulative incidence reached 11.2% (95% CI: 4–22.5%) and remained stable through day 60. In 2021 and 2022, mortality was absent until day 10, then increased to 8.8% and 2.6%, and remained unchanged through day 60 (95% CI: 1.9–22.3%; 0.6–8.2% for 2021 and 2022). These values indicate similar mortality profiles across pandemic years, with overlapping confidence intervals and delayed onset. The probability curves indicate that 2021 had the highest estimated mortality risk, followed by 2020 and 2022. These findings suggest that fatal outcomes were concentrated in the later stages of follow-up, potentially because of delayed clinical deterioration or post-hospital complications (Tables 10–12, Figure 4, and Supplemental Table 7).

## DISCUSSION

According to the results obtained, patients with severe dengue or with unusual clinical presentation tend to be younger and have higher mortality rates, which is consistent with the findings of other studies.^28^^,^^29^ In Colombia (2022), the highest incidence rates were observed in the age group of 10 to 14 years (539.1 cases per 100,000 inhabitants), followed by the 5 to 9 years group (475.0 cases per 100,000 inhabitants) and the 15 to 19 years age group (325.2 cases per 100,000 inhabitants). A study conducted in a high-complexity hospital in Cali (Colombia, 2013) revealed that severe forms of infection and mortality were more frequent in the pediatric population. Of 1,173 patients, 30% were under 13 years old; 42.9% had severe dengue, and 35.7% had dengue with warning signs.^30^ Similarly, in Cuba, dengue has affected both children and adults; however, ∼64% of the deceased were under 15 years old.^31^ Additionally, during the epidemic in Peru in 2023, there was a higher risk of developing severe plasma leakage and dengue shock in children; the pediatric population was the most affected by severe dengue.^32^

A similar pattern was observed in a tertiary hospital in Bangladesh in 2022, where the highest proportion of severe dengue cases was estimated among minors.^33^ During the 2019 dengue outbreak in India, a higher number of positive cases were observed in the age group of 21 to 30 years.^34^ Generally, cases are concentrated among young and mature adults because of comorbidities, transmission in the workplace or educational settings, and the frequency of travel and migratory processes.^33^ These results may be clinically valuable in determining the selection criteria for future interventions,^33^^,^^35^ as well as for paving the way for new studies that provide a more in-depth explanation of the reasons behind the occurrence of serious manifestations in the young population.

Various studies in Asia have revealed a consistent predominance of severe dengue cases in men,^33^^,^^35^^–^^37^ unlike in South America, where the number of severe dengue cases in women was similar to or higher than the number in men.^33^ The present investigation revealed both situations; although the majority of dengue cases involved women, the deceased and hospitalized patients were predominantly men. The potential differences between men and women could be related to women’s greater use of healthcare services.^33^ As for the severity of the infection in men, this could be partially explained by their occupational activities: men tend to work outdoors or move frequently, which increases their likelihood of contact with the vector and later opportunities for consultation.^33^^,^^37^ Likewise, there has been discussion of a greater inherent susceptibility in men, which explains their longer hospital stays compared with women. Immunological advantages associated with microRNAs linked to the X chromosome have been studied in women.^33^^,^^36^^,^^38^

The subsidized health insurance scheme covered the largest proportion of patients with severe dengue. This distribution may be linked to patients’ area of residence and other social determinants of health, as insurance type is closely associated with socioeconomic conditions. In this context, studies in Latin America have revealed an association between severe dengue and low-income populations (odds ratio: 1.28; 95% CI: 4.77–5.18).^39^ Additionally, severe dengue has been linked to delays in hospitalization after symptom onset, which reflects contextual barriers and limited accessibility to timely care.^33^ In the present study, most patients came from the urban areas of Cali. Beyond cultural factors related to risk perception and access barriers, it is essential to consider the urban environment as a more favorable habitat for *Aedes aegypti*, which may facilitate vector propagation within the territory.^33^

The opportunity for consultation reported in the present study, in general and by severity level, aligns with the 2022 Bangladesh study. In that study, an average of 5 days (SD ± 2.44 days) was estimated between symptom onset and hospitalization.^33^ The average delay in hospitalization was shorter for patients with classic manifestations and longer for those with severe dengue.^33^ Similarly, an association was observed between the length of patients’ hospital stays and the delay in hospitalization after symptom onset. These findings, along with those from the present study, suggest that early recognition of symptoms and timely therapeutic management can reduce clinical complications and shorten hospital stays.^33^

Significant differences were also found between comorbidities and severity levels. The authors of some previous studies have focused on analyzing the relationship between comorbidities and symptom worsening.^40^^,^^41^ The authors of one such paper analyzed complications in transplant patients, who took longer to normalize their platelet counts (6 ± 4.5 days; *P* <0.001). These patients, along with those suffering from chronic kidney disease, experienced worsening kidney function after a dengue infection.^39^^,^^40^ Another example involves patients with heart disease or myocarditis, in whom fluid leakage due to dengue can lead to heart failure. These patients present with tachycardia and early shock, conditions that can be fatal if not promptly detected and managed therapeutically.^41^ The present research revealed that chronic hematological diseases (mainly sickle cell anemia) and chronic kidney disease were associated with severe dengue.

Coinfections are also linked to severe dengue because of misdiagnosis or delayed treatment.^42^ An example is the potential cross-reaction between antibodies against DENV and severe acute respiratory syndrome coronavirus 2 (SARS-CoV-2), which may complicate the pathogenesis of dengue.^42^^,^^43^ In such coinfection, although patients exhibit thrombocytopenia, elevated transaminases, and increased C-Reactive protein levels, they also exhibit alterations in D-dimer and ferritin levels.^42^ Likewise, malaria coinfection impacts the clinical presentation of dengue, hematological parameters, the need for blood transfusion, and mortality rates. This complicates differential diagnosis and therapeutic approach procedures when evaluating febrile patients.^29^ Overall, the clinical presentation is more severe in cases of dengue coinfected with malaria, resulting in significantly more extended hospital stays and additional medical needs.^29^ Although Cali is not a district with endemic malaria transmission, 239, 345, and 334 cases were reported in 2020, 2021, and 2022, respectively. Most of these cases originated from other municipalities or departments. According to records from Cali’s District Secretariat of Public Health, only seven, two, and six cases, respectively, were reported as locally acquired during those years.^44^^–^^46^

Regarding hospitalization and ICU admission, a hospital stay of 2.0 days and an ICU stay of 4.5 days were estimated, similar to findings from other studies. In Malaysia (2019), patients hospitalized for dengue had an average hospital stay of 3.43 days (SD ± 2.08 days) and a median of 3 days (IQR = 7 days).^47^ However, in southern India, a tertiary care facility reported an average hospital stay of 4.9 days (SD ± 2.4 days).^48^ The Bangladesh study (2022) revealed an average stay of 4.9 days (SD ± 1.65 days). In the present study, only 29.9% of patients remained in the hospital for more than 5 days.^33^ By age category, adults over 60 years averaged a 4-day stay (95% CI: 4.00–4.00 days); those between 41 and 60 years averaged a 5.1-day stay (95% CI: 4.34–5.83 days); adults between 18 and 40 years, averaged a 5-day stay (95% CI: 4.77–5.18 days); and children under 18 years averaged a 4.4-day stay (95% CI: 4.34–5.83 days).^33^ Middle-aged patients experienced longer hospitalizations due to multiple disorders, a relatively weaker immune system compared with younger individuals, and delayed consultation and hospitalization.^33^

In terms of mortality, patients in the present study succumbed to multiple organ failure, acute myocardial infarction, cerebral herniation, intracranial hemorrhage, and septic shock. These outcomes are consistent with reports in the literature indicating that cases with warning signs and severe dengue can reach a mortality rate of up to 20%, influenced by various factors.^28^ In a northern India tertiary hospital, ∼6% of patients treated between 2011 and 2016 died; the majority were men, with an average age of 31.6 years (SD ± 14 years) and diagnosis with severe dengue upon admission (76.5%).^49^ Among these lethal cases, dyspnea (47%), tachypnea (86.7%), leukocytosis (58.8%), elevated urea (80%), and elevated serum creatinine (52.9%) at admission, as well as shock during hospitalization (58.8%), increased the risk of death.^49^ This finding underscores the importance of the early identification of warning signs and comorbid conditions that could exacerbate the patient’s condition.^28^

By epidemiological year, although epidemic years exhibit a higher number of cases, the case fatality rate and the incidence of ICU admission are greater during endemic periods (2021 and 2022). To interpret the study results by epidemiological year, data were analyzed in the context of the SARS-CoV-2 pandemic because concurrent or overlapping infections can alter the typical clinical course, severity, or outcome of each infection.^42^^,^^50^ Coinfection with SARS-CoV-2 and DENV has been linked to poorer morbidity and mortality outcomes.^51^ Patients typically present with fever, dyspnea, headache, cough, and signs of thrombocytopenia, lymphopenia, elevated transaminases, and leukopenia.^50^ Complications include septic shock, acute respiratory distress syndrome, and multiple organ failure.^50^^,^^52^ The authors hypothesize that the coronavirus disease 2019 pandemic also affected the hospitalization duration of dengue patients. In a Singapore tertiary general hospital, there was a mid-2020 surge in dengue hospitalizations, with an average stay of 8.35 days (SD ± 6.53 days) attributed to isolating febrile patients while awaiting SARS-CoV-2 polymerase chain reaction test results and potential complications.^52^^,^^53^

The consultation opportunity and hospitalization delays during these years must also be considered to understand the increased complications observed in the present study in 2020 and 2021. During this period, Colombia, like other countries, implemented restrictions such as collective isolation, individual responsibility isolation, and prioritization of health services to manage the pandemic; these measures led to increased consultation opportunities and delayed hospitalization for dengue cases,^53^ as well as heightened vector density during lockdowns due to insufficient vector control programs.^54^ Potential contributing factors include the public’s reluctance to seek healthcare services, the reallocation and demand for health professionals, and the underreporting of arboviruses during the pandemic.^55^

Another factor is the increasing incidence of DENV, driven by the expansion of the *Aedes aegypti* and *Aedes albopictus* vectors.^56^ The enhanced fitness of circulating DENV serotypes with specific virulence factors, influenced by changing environmental conditions and host immunity, impacts the infection rate and risk of severe dengue.^52^ Although information on dengue serotypes could not be gathered in the present study, evidence suggests that the coexistence of multiple serotypes exacerbates the condition and prognosis.^34^^,^^49^ In India, hyperendemicity with concurrent infections of two or more serotypes has been linked to dengue severity.^34^ Specifically, the DENV-2 serotype has been prevalent in secondary infection among Vietnamese adults and patients with severe disease characterized by pleural effusion and low platelet count.^57^ Dengue virus 2 infections required more fluid expansion, longer hospital stays (5.1 days; SD ± 2.8 days), and ICU admissions. According to reports from the INS, sustained circulation of DENV serotypes DENV-1, DENV-2, and DENV-3 was detected between 2019 and 2022, with a reemergence of DENV-4 in 2022. In Valle del Cauca (a department in southwestern Colombia), DENV-1 predominated in 2019, followed by an increase in DENV-2 circulation in 2020 and 2021 and concurrent circulation of DENV-1, DENV-2, and DENV-4 in 2022.^58^^,^^59^ Although virus variability is not the primary driver of large outbreaks, DENV lineages can have variable phenotypes that affect virulence, transmissibility, and immune evasion.^60^ This finding highlights the need for future research that includes this variable to complement the analyses in the present study.

### Strengths and limitations of the present study.

The current study was conducted using data collected from public and private health institutions and had an adequate sample size. Although data were collected from three institutions with different levels of care (primary and tertiary, public and private), the study design was based on a single cohort with systematic follow-up of the clinical course of each patient diagnosed with dengue. Although tertiary institutions are expected to focus on more severe cases with higher mortality rates, the main objective was to observe the clinical evolution and the occurrence of severe and fatal outcomes, regardless of the point of entry into the health system. In this sense, although patients could be admitted at different levels of severity depending on the type of institution, their clinical condition and the probability of complications were evaluated consistently throughout the follow-up, thereby maintaining the internal validity of the study.

The inclusion of multiple institutions enabled the capture of a broader range of clinical trajectories and care contexts, enriching the understanding of dengue behavior under real-world conditions. This facilitated an exploratory subgroup analysis to control for disease severity and socioeconomic variables that could influence the results. However, the characteristics of each institution, such as its role as a reference, installed capacity, and population profile, may influence the distribution of observed outcomes. Analyzing this heterogeneity in depth is recommended in future studies.

Additionally, not all cases were virologically confirmed, reflecting the operational conditions of the healthcare system in Cali, an endemic area where clinical assessment is the primary diagnostic approach in settings of high viral circulation. Furthermore, in the present study, obtaining information on dengue serotypes was not possible due to administrative constraints. Such data could have enriched the analysis and discussion of the findings because specific serotypes are associated with distinct clinical manifestations. Regarding immunological status, unfortunately, serological information to distinguish between primary and secondary infections was unavailable, as seroprevalence studies are not routinely conducted in clinical care. This limitation restricts the ability to interpret, in greater depth, the factors that may influence the observed clinical severity. Nevertheless, the cohort design enabled systematic follow-up of patients’ clinical courses, providing valuable evidence on outcome variability, even in the absence of universal virological confirmation, serotype identification, or immunological characterization.

Because of the study’s design, although it is representative of a finite population, the results are not generalizable to non-institutionalized populations or populations with characteristics that differ from those of the study’s population.

## CONCLUSION

The present study enables the identification of possible subgroups that may require specific approaches and generate inputs for the challenges of care and treatment of patients with dengue in the short and medium term. Therefore, age is a risk factor for the development of complications and serious symptoms, underscoring the need to enhance surveillance in minors and young adults, as well as to prioritize them in the implementation of preventive measures. It is also advisable to monitor individuals with chronic hematological diseases, kidney disease, severe cardiovascular disease, and coinfections early on, utilizing risk communication strategies in the community and among health personnel. Moreover, based on the findings of the present study, it is crucial to raise awareness of the importance of timely consultation, as any delay beyond 5 days after symptom onset can extend recovery time, exacerbate complications, and increase the likelihood of ICU admission and mortality within 10 days of seeking healthcare services.

During endemic periods, the incidence of ICU admissions and case fatality increased. These observations suggest more severe admission conditions or greater complications during endemic periods compared with epidemic periods. Endemic periods remain a concern in terms of ICU admissions and case fatality. The present study represents an initial step in analyzing the clinical course of dengue across different temporal heterogeneities or endemic periods of the virus. Overall, the study results contribute to a better understanding of the factors that lead to severe and fatal outcomes. Accurate identification of demographic and clinical characteristics, effective risk communication, and timely, appropriate clinical management tailored to temporal and contextual needs are vital to reducing hospitalization rates and fatalities. Analysis of the probabilities of presenting specific moderate and severe outcomes by degree of endemicity provides a clearer view of the relationship between these variables and the dynamics of transmission and the evolution of the condition. Therefore, governments cannot limit measures to prevent the spread of the disease under “normal” or expected circumstances in endemic territories, especially when new prevention methods are available, such as vaccination.

Future multivariate analyses in expanded or multicenter cohorts are recommended to enable a more in-depth exploration of the interaction among clinical, immunological, demographic, and contextual factors.

## Supplemental Materials

10.4269/ajtmh.25-0158 Supplemental Materials
