# Supplementary material for: Dengue’s Clinical Course in One of Colombia’s Most Endemic Areas: Impact of Endemic and Epidemic Periods
Source: Am J Trop Med Hyg. 2026 Feb 26;114(4):724–37. doi: 10.4269/ajtmh.25-0158 (PMC13045547; doi:10.4269/ajtmh.25-0158)
Supplement: Supplemental Materials [file tpmd250158.SD1.pdf]

SUPPLEMENTARY MATERIAL

Table 1S. Criteria used for the classification of Dengue, Santiago de Cali, 2019-2022.

**Categories**	**Considerations**
**Dengue without warning signs**	
Fever	Those cases that had these conditions, or did not have any alarm criteria or serious criteria, were considered *Dengue without alarm signs*.
Headache	
Retroocular pain	
Myalgias	
Arthralgias	
Eruption	
Rash	
**Dengue with warning signs**	
Abdominal pain	-
Vomit	-
Diarrhea	-
Drowsiness or irritability	This sign included altered behavior and altered state of consciousness.
Hypotension	It was defined in terms of: systolic pressure < 90 mm Hg or diastolic pressure < 60 mm Hg.
Hepatomegaly	-

Mucous membrane hemorrhage	This sign included hematuria, petechiae, ecchymoses, hematemesis, vaginal bleeding, epistaxis, and gingivorrhagia.
Hypothermia	Due to the amount of missing data, this sign was omitted.
Fluid buildup	This sign included ascites.<br><br>Pulmonary edema was not included because all those who had this condition had target organ involvement; therefore, they were classified as *severe dengue*.
**Severe dengue**	
Extravasation	-
shock	-
Myocarditis	-
Encephalitis	-
Liver involvement	-
Renal failure	-

Table 2S. Variables with the highest proportion of missing data, Santiago de Cali, 2019-2022 (n = 1038).

Variables*	Missing data (%)
Temperature	79
Albumin	77
Fever duration	52
Creatinine	42
AST transaminases	34
ALT transaminases	33
No. days of disability	26
Location	25
Systolic blood pressure	7
Diastolic blood pressure	7

*First 10 variables with the highest percentage of missing data.

Table 3S. Regression model of factors associated with severe dengue and dengue with warning signs, Santiago de Cali, 2019-2023.

**Variables***	**Dengue with warning signs**	**Pr(>|z|)**	**Severe dengue**	**Pr(>|z|)**
(Intercept)	**2.03**	**0.001**	**0.07**	**<0.001**
MALE Sex	0.98	0.903	1.38	0.187
Age	**0.98**	**<0.001**	1	0.745
Diabetes YES	**0.26**	**0.047**	0.53	0.537
UNINSURED Regime	1	1	0.73	0.537
SUBSIDIZED Regime	1.03	0.921	0.93	0.88
Arterial Hypertension YES	1.19	0.636	**2.52**	**0.097**
Chronic Kidney Disease YES	1.3	0.822	**6.44**	**0.092**
Serious Cardiovascular Disease YES	**3.57**	**0.063**	1.73	0.562
Acid-Peptic Disease YES	1.29	0.631	**0.08**	**0.009**
Autoimmune Disease YES	0.56	0.679	0.6	0.903
Seizure Syndromes YES	2.33	0.54	0.65	0.917
COPD YES	0.45	0.562	4.22	0.249
COVID-19 YES	1.52	0.51	0.98	0.984
Re-entry to the Institution YES	0.65	0.115	**0.3**	**0.047**
Stay in the service	**1.07**	**0.01**	**1.07**	**0.019**
Chronic Hematological Diseases YES	0.72	0.621	1.15	0.855
Consultation opportunity	1.05	0.163	1.05	0.244

***Reference values/categories:**

Severity: Dengue with warning signs.

Sex: Female.

Institution: Clínica Imbanaco

Regime: Contributory.

Comorbidities: No.

Note: To identify the factors that might differentiate the probability of developing any type of dengue (dengue without warning signs, dengue with warning signs, and severe dengue), according to the baseline conditions and sociodemographic characteristics of the patients, a multinomial logistic regression model was estimated, using dengue without warning signs as the reference category. The odds ratios and the Pr (>|z|) (p-value associated with the z value of a predictor variable in logistic regression) were calculated to identify the variables that could be protective factors (values less than 1) or risk factors (values greater than 1) associated with dengue with warning signs or severe dengue, compared to the reference category.

Table 4S. Operational characteristics of the regression model of factors associated with severe dengue and dengue with warning signs, Santiago de Cali, 2019-2023.

Clinical feature	Dengue without warning signs	Dengue with warning signs	Severe dengue
Sensitivity	0.18	0.82	0.88
Specificity	0.94	0.58	0.90
Positive predictive values	0.52	0.64	0.76
Negative predictive values	0.76	0.78	0.95
F1	0.26	0.72	0.81

Table 5S. Operational characteristics of the regression model of factors associated with severe dengue and dengue with warning signs, Santiago de Cali, 2019-2023.

**Variables n (%)**	**DWWS n = 87**	**DWS n = 154**	**SD n = 9**	**DWWS n = 24**	**DWS n = 52**	**SD n = 239**	**DWWS n = 166**	**DWS n = 281**	**SD n = 26**	**Total n = 1038**	**p-value**
	**Institution FLHI (public) n = 250**			**Institution TLHI (public) n = 315**			**Institution TLHI (private) n = 473**				
**Final condition**											**< 0.001**
Alive	87 (100)	153 (99.35)	9 (100)	24 (100)	52 (100)	227 (94.98)	166 (100)	280 (99.64)	23 (88.46)	1021 (98.36)	
Dead	0 (0)	1 (0.65)	0 (0)	0 (0)	0 (0)	12 (5.02)	0 (0)	1 (0.36)	3 (11.54)	17 (1.64)	
**Hospitalization**											**< 0.001**
Yes	28 (32.18)	67 (43.51)	7 (77.78)	20 (83.33)	38 (73.08)	212 (88.70)	46 (27.71)	150 (53.38)	19 (73.08)	587 (56.54)	
No	59 (67.82)	87 (56.49)	2 (22.22)	4 (16.67)	14 (26.92)	27 (11.30)	120 (72.29)	131 (46.62)	7 (26.92)	451 (43.44)	
**ICU admission**											**< 0.001**
Yes	0 (0)	0 (0)	0 (0)	1 (4.17)	6 (11.54)	49 (20.50)	4 (2.41)	26 (9.25)	7 (26.92)	93 (8.96)	
No	87 (100)	154 (100)	9 (100)	23 (95.83)	46 (88.46)	190 (79.50)	162 (97.59)	255 (90.75)	19 (73.08)	945 (91.04)	
**Incidence of hospitalization***	32.18	43.51	77.78	83.33	73.08	88.70	27.71	53.38	73.08	56.54	-

**Incidence of ICU admission***	0	0	0	4.17	11.54	20.50	2.41	9.25	26.92	8.96	-
**Lethality**	0	0.65	0	0	0	5.02	0	0.36	11.54	1.64	-

FLHI (public): first-level public health institution.

TLHI (public): third-level public health institution.

TLHI (private): third-level private health institution.

DWWS: Dengue without warning signs.

DWS: Dengue with warning signs.

SD: Severe dengue.

*Study population at risk (multiplication coefficient: 100).

Table 6S. Assumption of proportional risks of hospitalization, ICU admission and mortality per year, Santiago de Cali, 2019-2022.

Variable	Decision	p value
Mortality	Is fulfilled	0.14
Admission Hospitalization	Is fulfilled	0.72
ICU admission	Is fulfilled	0.19

Table 7S. Difference in outcomes of hospitalization, ICU admission and mortality by chronological year (Hazard ratio), Santiago de Cali, 2019-2022.

Variable	Decision	p value
Mortality	There are no differences	0.61
Admission Hospitalization	There are no differences	0.74
ICU admission	There are no differences	0.24
